# Pharmacodynamics, safety, and immunogenicity of Pelmeg^®^, a pegfilgrastim biosimilar in healthy subjects

**DOI:** 10.1002/prp2.507

**Published:** 2019-08-13

**Authors:** Hendrik Wessels, Dirk Lehnick, Josef Höfler, Ruediger Jankowsky, Paul Chamberlain, Karsten Roth

**Affiliations:** ^1^ Cinfa Biotech GmbH (now part of the Mundipharma network of independent associated companies) Munich Germany; ^2^ University of Lucerne Lucerne Switzerland; ^3^ Staburo GmbH Munich Germany; ^4^ NDA Advisory Services, Ltd, Grove House Leatherhead Surrey UK

**Keywords:** biosimilars, filgrastim, highly similar, myelosuppressive chemotherapy, neutropenia, oncology, pegfilgrastim, supportive care

## Abstract

A pharmacodynamics (PD) and immunogenicity study was conducted to investigate biosimilarity of Pelmeg^®^, a pegfilgrastim biosimilar to EU‐authorized Neulasta^®^. The multiple‐dose, randomized, double‐blind, two‐sequence, and three‐period cross‐over study comprised 96 healthy male subjects, receiving Pelmeg (Test [T]) and Neulasta (Reference [R]) in a sequential manner (T‐T‐R vs R‐R‐T). Subjects were dosed with 3 mg pegfilgrastim, as this dose was previously shown to be in the ascending part of the dose‐response curve for PD. The primary PD endpoint was the area under the effect curve (AUEC_0‐last_) for absolute neutrophil count (ANC). The primary immunogenicity endpoint was proportion of anti‐drug antibody (ADA)‐positive subjects at the end of Period 2 (ie, after administration of two doses of the same study drug). Comparability was demonstrated for the PD endpoint, with the geometric mean ratio (T/R) of AUEC_0‐last_ being 101.59%, with a corresponding 95% CI of [99.58; 103.63]. Of note, when using tighter acceptance limits (90.00%‐111.00%), comparability between test and reference was shown as well. Only two confirmed ADA positive samples were detected, one after treatment with Pelmeg and one after Neulasta. These had a low ADA titer, no filgrastim reactivity, and no neutralizing capacity. No clinically meaningful differences in safety between Pelmeg and Neulasta were observed. Overall, the results from this study confirmed the biosimilarity of Pelmeg and Neulasta for PD and immunogenicity, as shown already at the bioanalytical level and in the pivotal PK/PD study with Pelmeg.

**Abbreviations**
ADA: anti‐drug antibody
AEs: adverse events
ANC: absolute neutrophil count
AUEC: area under the effect curve
BMI: body mass index
CV: coefficient of variation
ECL: electroluminescence
ELISA: enzyme‐linked immunosorbent assay
EMA: European Medicines Agency
G‐CSF: granulocyte colony‐stimulating factor
IVD: in vitro diagnostic
PD: pharmacodynamics
PEG: polyethylene glycol
PK: pharmacokinetic
PTs: preferred terms
QC: quality control
SAEs: serious adverse events
SOC: System Organ Class
TMB: tetramethylbenzidine

## 1 INTRODUCTION

Chemotherapy impacts rapidly dividing cells by directly causing cell death and slowing or stopping proliferation. Due to these effects, many chemotherapy regimens are associated with myelosuppression, resulting in reduced production of neutrophils (and also other blood cells like erythrocytes and thrombocytes). Often such hematological toxicities limit the delivery of the planned dose and intensity of chemotherapy, which is crucial for tumor control and patient survival. In clinical practice, neutropenia is the main limiting factor for the applicability of chemotherapy.^1^

Thereby, both the duration of Grade 4 neutropenia (defined as absolute neutrophil count [ANC] of <0.5 × 10^9^/L) and the depth of the nadir after chemotherapy are correlated with the development of infectious complications.^2^ Thus, an important goal in oncological practice is the prevention of neutropenia when administering chemotherapy.

Filgrastim is a recombinant human granulocyte colony‐stimulating factor (G‐CSF), which stimulates the production of neutrophil precursors, enhances the function of mature neutrophils, and ameliorates neutropenia and its complications.^3^ Pegfilgrastim is a pegylated form of filgrastim, developed to increase its half‐life. Pegfilgrastim retains the same biological activity as filgrastim and binds the same G‐CSF receptor. A once‐per‐chemotherapy‐cycle administration of pegfilgrastim was shown to be sufficient to reduce the duration of severe neutropenia as effectively as daily treatment with filgrastim.^4^

The efficacy and safety of pegfilgrastim (Neulasta) for prevention of chemotherapy‐induced neutropenia was demonstrated in two pivotal Phase 3 studies,^2^, ^5^ leading to regulatory approval of Neulasta in the US and the EU.

Pelmeg (development code B12019) was developed as a biosimilar to Neulasta. A comprehensive analytical, functional, and preclinical comparability program has demonstrated a high degree of similarity of Pelmeg to Neulasta.^6^ In the clinical development program, two comparative studies have been conducted to investigate differences between Pelmeg and Neulasta.

The first and pivotal study (B12019‐101) has demonstrated pharmacokinetic (PK) and pharmacodynamic (PD) comparability to Neulasta while using the clinical dose of 6 mg (study B12019‐101; manuscript submitted for publication). The second and supportive study (B12019‐102) mainly aimed to confirm PD similarity between Pelmeg and Neulasta at a more sensitive dose, and to investigate any potential differences in immunogenicity, which is considered as a general safety concern common to all therapeutically applied proteins.

Various factors were considered when designing this study.

The study was conducted in healthy subjects. Compared to cancer patients receiving chemotherapy, healthy subjects lack comorbidities and comedications, and are not immunosuppressed. Thus, they represent the most sensitive study population for conducting the PD comparison. The use of a sensitive population is recommended by the Guideline on similar biological medicinal products containing biotechnology‐derived proteins as active substance: nonclinical and clinical issues (EMEA/CHMP/BMWP/42832/2005 Rev 1). Also, regarding the assessment of potential immunogenicity of pegfilgrastim, healthy subjects are considered more sensitive than cancer patients, as the latter have a compromised immune system.

In both healthy and patient populations, the mechanism of action of pegfilgrastim is the same, whereby pegfilgrastim elicits its effects on hematopoietic cells by binding to specific cell surface receptors stimulating proliferation and differentiation of committed progenitor cells of the granulocyte‐neutrophil lineage into functionally mature neutrophils. Because the bone marrow in a healthy subject population is functionally unimpaired (in comparison with patients undergoing myelosuppressive chemotherapy), the bone marrow of this subject population is expected to be more responsive to stimulation with G‐CSF.^7^

The primary PD parameter ANC is an accepted surrogate marker and can be related to patient outcome to the extent that demonstration of similar effect on the PD marker will ensure a similar effect on the clinical outcome (Guideline on similar biological medicinal products containing biotechnology‐derived proteins as active substance: nonclinical and clinical issues, EMEA/CHMP/BMWP/42832/2005 Rev 1). The 3 mg dose was chosen as it was shown to be in the ascending part of the dose‐response curve for PD,^8^, ^9^ and thus being more sensitive to detect potential differences in PD between Pelmeg and Neulasta. The cross‐over design helped to minimize variability of the PD parameter.

In this study subjects were dosed twice with Pelmeg or Neulasta, as the likelihood to initiate an immuneresponse, for example, anti‐drug antibodies (ADAs) generation is higher after multiple dosing compared to single dose. For a product such as pegfilgrastim, for which there is no identified clinically impactful immunogenicity, it is not feasible to prespecify an acceptable margin of difference in the incidence of treatment‐related ADA across the biosimilar and reference product treatment arms for the purpose of the biosimilarity assessment. Therefore, clinical evaluation of relative immunogenicity involved repeated administration in a parallel‐group design using fully immune competent healthy volunteers to provide the most sensitive test conditions to detect a potential difference in the relationship between ADA signals and PK and PD. This enabled interpretation of the clinical impact, if any, of anti‐pegfilgrastim ADA.

Objectives of this study were to demonstrate PD comparability based on area under the effect curve (AUEC_0‐last_) for ANC, and to compare the immunogenicity of Pelmeg and Neulasta, when administered at doses of 3 mg. Overall results from this study, as presented here, confirm the similarity to EU‐authorized Neulasta with regard to PD and immunogenicity.

## 2 MATERIALS AND METHODS

This randomized, double‐blind, multiple‐dose, three‐periods, and two‐sequences cross‐over study in healthy subjects was conducted at two study sites in Germany, between August 2016 and February 2017.

The study was registered with EudraCT (number 2015‐005022‐19) and conducted in accordance with the International Conference on Harmonisation Guideline for Good Clinical Practice E6, the European Clinical Trial Directives 2001/20/EC and 2005/28/EC, and applicable national and local regulatory requirements. The aspects of the study concerned with the investigational medicinal product met the requirements of EU Good Manufacturing Practice. The protocol and informed consent form were reviewed and approved by relevant ethics committees prior to implementation. Written informed consent was obtained from all subjects prior to screening.

### 2.1 Study population

Healthy male subjects (as determined by medical history, physical examination including vital signs, electrocardiogram [ECG] and clinical laboratory testing), aged 18‐55 years, with a body mass index (BMI) between 20.0 and 30.0 kg/m^2^ (inclusive), and a weight between 60 and 100 kg (inclusive) were eligible to be included in the study. All subjects were to comply with the contraception requirements as specified in the protocol. Subjects were excluded if they had been previously treated with pegfilgrastim, or if they had known ADAs to filgrastim, pegfilgrastim, or polyethylene glycol (PEG).

### 2.2 Study design

A total of 96 subjects (80 evaluable subjects plus 16 subjects accounting for possible drop‐outs) were allocated to receive Pelmeg (T) or Neulasta (R) in a two‐treatment, two‐sequence, and three‐period cross‐over design. The main purpose of the design and size of the study was to investigate potential major differences in immunogenicity between Pelmeg and Neulasta. Forty evaluable subjects per sequence were considered to provide an appropriate precision of estimates for the proportions of ADA‐positive subjects.

The given sample size also supported the assessment of biosimilarity for the PD endpoint, AUEC_0‐last_ for ANC. Assuming an expected true test/reference ratio of 0.95‐1/0.95 and equivalence limits of 80.00%‐125.00% for the 95% CIs, 80 evaluable subjects provided 90% power to lie within the acceptance ranges, as long as the intraindividual coefficient of variation (CV) did not exceed 40%.

**Figure 1:**
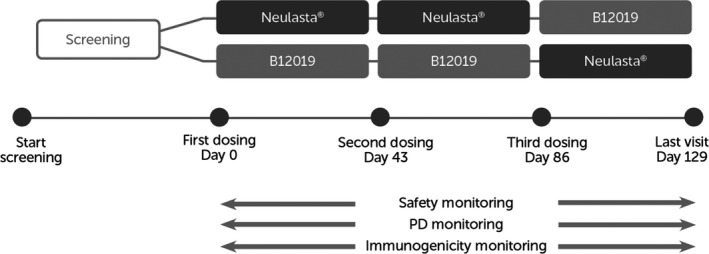
Study design B12019‐102

The study design is shown in Figure 1.

Subjects were screened within 28 and 2 days prior to administration of study drug. Eligible subjects were admitted to the study site and remained hospitalized until Day 5, while ambulatory visits were performed after Day 5 until Day 43 of each period. Each subject participated in three study periods. Subjects were randomized in a 1:1 ratio to sequentially receive T‐T‐R or R‐R‐T. Dosing was separated by a wash‐out period of at least 6 weeks (maximum 8 weeks), corresponding to approximately 15 half‐lives. Study drugs were administered as subcutaneous (s.c.) injections into the abdomen, at a dose of 3 mg (Pelmeg: 3 mg/0.3 mL, batch number 9201516002, Cinfa Biotech SL, Spain, Neulasta: 3 mg/0.3 mL, batch number 1061466C, Amgen Europe BV, The Netherlands).

### 2.3 Endpoints and statistical analysis

The primary immunogenicity endpoint was proportion of ADA‐positive subjects at the end of Period 2, as detected by a confirmatory assay. The primary immunogenicity analysis was performed on the safety set, defined as all subjects who received at least one dose of study drug. For the primary analysis, proportions of ADA‐positive subjects at the end of Period 2 were calculated and presented with corresponding 95% CIs per treatment. Furthermore, the difference of proportions of ADA‐positive subjects between treatments was calculated and presented with corresponding 95% CIs.

The primary PD variable was AUEC_0‐last_ for ANC. The primary PD analysis was performed on the model‐based PD set, defined as all subjects with reliable PD data for all three study periods. AUEC_0‐last_ was regarded as unreliable if more than three consecutive or nonconsecutive samples are missing or if the ANC values were quantifiable for fewer than five time points. To demonstrate PD comparability of Pelmeg vs Neulasta the primary PD parameter was calculated. Pelmeg and Neulasta were assumed to be biosimilar regarding PD if the 95% CI of the test/reference ratio lay within the acceptance interval of 80.00%‐125.00%. The 95% confidence limits were calculated based on the antilogs of the least square means and mean square error from a GLM analysis of variance with sequence, subjects within sequence, period and treatment as fixed effects on log‐transformed AUEC_0‐last_ of ANC data. In order to achieve a better approximation to a normal distribution, PD parameters related to concentrations (such as AUEC_0‐last_) were logarithmically transformed before analysis.

Secondary PD variables were maximum effect (*E*_max_) and time to *E*_max_ (*t*_max, E_) of ANC, and CD34^+^ counts. They were evaluated descriptively, based on the PD set (defined as all subjects who had evaluable PD data from at least one study period).

Secondary PK variables were area under the concentration curve from time 0 to 120 hours (AUC_0‐120 h_), maximum concentration (*C*_max_), and time to *C*_max_ (*t*_max_). They were evaluated descriptively, based on the PK set.

Safety variables included adverse events (AEs), local tolerability, physical examinations, vital signs, 12‐lead ECG, and laboratory safety assessments. Safety results were summarized descriptively.

### 2.4 Bioanalysis

#### 2.4.1 Analysis of ANC and CD34

Blood samples for determination of ANC were collected during the in‐patient phase, predose and up to 96 hours postdose, and during the ambulatory visits in each period. Determination of ANC from whole blood was performed by fluorescent flow cytometry, using the automated hematology analyzer XT‐2000i (SYSMEX) and reagents. Before samples from the clinical study were analyzed, quality control (QC) samples (including three concentration levels) were measured on each day of the analytical performance. Only after acceptance of QC samples, study samples were analyzed. The method was validated by the provider.

Blood samples for determination of CD34^+^ were collected on Day 1 (predose), and between Day 3 and Day 10 postdose. The frequency of CD34^+^ cells from whole blood was determined with a flow‐cytometry based assay, using the BD Bioscience Stem Cell Enumeration Kit in combination with the FACS Canto Clinical Software. The kit is an FDA‐cleared in vitro diagnostic IVD) test which meets the ISHAGE Guidelines.^10^ The sensitivity of the assay was determined as 2.7 CD34^+^ cells/µL.

#### 2.4.2 Analysis of ADAs

Blood samples for ADA analysis were obtained on Day 1 predose, Days 8, 15, 22, 29, and 43 of each period.

Anti‐pegfilgrastim antibodies in serum were detected with an immunoassay using electroluminescence (ECL); detergents were excluded from the assay and wash buffers to optimize sensitivity to detect antibodies reactive with both the filgrastim and PEG moieties of pegfilgrastim. The testing concept involved a multi‐tiered approach. Initially, samples were subjected to a run‐specific screening assay. If a sample result exceeded the cut point of the screening assay, then the sample was considered as ADA‐reactive and was advanced to the next tier. Otherwise, the sample was considered negative, and no further tests were required on the sample. All samples that were ADA‐positive in the screening assay were subsequently tested in a confirmatory assay. In the confirmatory assay, samples were tested in parallel with four different competitive inhibitors (Pelmeg, Neulasta, Filgrastim, PEG6000). Samples that gave a percentage inhibition value equal to or greater than the confirmatory cut point were classified as positive for the respective competitive inhibitor. Relative sensitivity was demonstrated and controlled using a rabbit anti‐Pelmeg whole molecule affinity‐purified IgG antibody reagent (custom reagent prepared by Squarix GmbH), in combination with a mouse monoclonal anti‐PEG IgM positive control antibody reagent (ANP Technologies Cat. No. ANPEG‐1); the relative detection sensitivities were 22 ng/mL for the anti‐Pelmeg IgG and 114 ng/mL for the anti‐PEG IgM. A conservative test strategy was applied to classify samples as ADA positive if any reactivity with Pelmeg, Neulasta, filgrastim, or PEG6000 was detected in a confirmatory assay. All confirmed positive samples were further characterized for ADA titer in a ligand‐binding assay format and for neutralizing capacity in a cell‐based assay (NSF‐60 assay).

The methods were developed in accordance with the EMA Guideline on immunogenicity assessment of therapeutic proteins (EMEA/CHMP/BMWP/14327/2006 Rev 1, May 2017).

#### 2.4.3 Analysis of pegfilgrastim concentrations

Blood samples for PK analysis were collected predose and up to 120 hours postdose.

Pegfilgrastim concentrations in serum were determined using a quantitative enzyme‐linked immunosorbent assay (ELISA) technique. The assay employed components from the R&D Systems (Biotechne AG, Switzerland) Human G‐CSF DuoSet ELISA kit. Microplates are coated with mouse anti‐human G‐CSF capture antibody which binds the G‐CSF in the sample. After the analyte is bound it is detected using a biotinylated goat anti‐human G‐CSF detection antibody. The bound capture antibody is then by binding of streptavidin‐horseradish‐peroxidase (HRP), which in turn enzymatically catalyses tetramethylbenzidine (TMB) conversion.

The determination was carried out over an expected calibration range of 0.20‐8.00 ng/mL (samples above the calibration range could be diluted up to 400‐fold). The method was validated in accordance with the European Medicines Agency (EMA) Guideline on Bioanalytical Method Validation (2011)^11^ and the FDA Draft Guidance for Industry on Bioanalytical Method Validation (2001).^12^

### 2.5 Compliance with design and statistical analysis requirements

The study was designed to enrol equal subject numbers for each treatment sequence, and subjects were randomized in a 1:1 ratio to the sequences T‐T‐R or R‐R‐T. Inclusion and exclusion criteria were predefined in the protocol. As there was a visible difference between the syringes for the test and reference products, drug administrations were performed by an unblinded team of medics and medically trained staff members, who were not involved in any further study activities, and in a way that the subjects remained blinded. Subjects, investigator staff, persons performing the assessments or being responsible for determining dosing regimen and staff of the sponsor or data analysts, remained blinded from the time of randomization until database lock.

## 3 RESULTS

### 3.1 Demographics and baseline characteristics

A total of 96 subjects were randomized and enrolled in the study (48 subjects for each treatment sequence). Of these, 47 were treated with the sequence RRT and 48 were treated with the sequence TTR. One subject, randomized to sequence RRT, was treated in an incorrect treatment sequence, due to administration of study drug for Period 3 in Period 1, resulting in an actual treatment sequence of TRR. This subject was analyzed “as treated.” Of the 96 subjects who received study medication, 12 discontinued the study prematurely (four subjects due to AE, six subjects withdrew consent [however, five of these agreed to return for the follow‐up visit], and two subjects due to nonallowed procedures). A total of 84 subjects completed all three study periods.

All subjects who received a dose of study medication were included in the safety/PK set. All subjects who had evaluable PD data from at least one study period were included in the PD set. For three subjects, no PD profile from at least one period could be calculated, as the subjects discontinued during period 1; these subjects were excluded from the PD set.

The model‐based PD set, used for the primary PD analysis, included only subjects with reliable data from all three study periods. Analysis sets are shown in Table 1.

**Table 1:** Analysis sets

	Treatment sequence		Total
	R‐R‐T	T‐T‐R	
Safety/PK set	48^a^	48	96
PD set	46^a^	47	93
Model‐based PD set	41	41	82
Analysis set for ADA frequencies	47^a^	48	95

Abbreviations: ADA, anti‐drug antibody; PD, pharmacodynamic; PK, pharmacokinetic; R, reference; T, test.

a The subject randomized to sequence RRT was treated with study drug for Period 3 in Period 1, leading to an incorrect treatment sequence of TRR. This subject was excluded from the analysis of ADA frequencies, as the treatment was incompatible with the intended study design.

Demographics and baseline characteristics are shown in Table 2 for the primary analysis set (model‐based PD set).

**Table 2:** Demographics and baseline characteristics

	N = 82
Age (years)	
Median	44
Min; max	21, 55
Weight (kg)	
Median	80.3
Min, max	62.4, 99.5
Height (cm)	
Median	180
Min; max	161, 194
BMI (kg/m^2^)	
Median	25.3
Min; max	20.0, 30.0
Race n (%)	
White	78 (95.1)
Asian	1 (1.2)
Black	3 (3.7)
Other	0 (0.0)
Smoking status n (%)	
Yes	13 (15.9)
No	69 (84.1)

Note

All subjects in this study were male. Thus, subject distribution by sex is not shown.

Abbreviations: BMI, body mass index; Max, maximum; Min, minimum; N, number of subjects.

### 3.2 Pharmacodynamics

Results are presented for the primary analysis set, the model‐based PD set. This set includes only subjects with reliable data from all three study periods.

**Figure 2:**
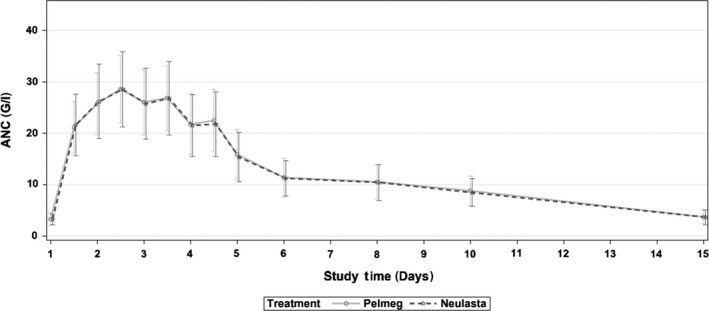
Mean (SD) ANC values until Day 43 (model‐based PD set, N = 82)

Mean ANC values after administration of Pelmeg and Neulasta are shown in Figure 2. ANC profiles were very similar after administration of Pelmeg and Neulasta. Starting from similar predose levels (around 3 G/L), comparable increases in mean ANC were observed. Peak levels were reached at around 36 hours postdose and decreased thereafter. Predose level was reached again on Day 22.

Results for the statistical analysis of the primary PD parameter are shown in Table 3. The geometric mean ratio of AUEC_0‐last_ was about 100% and the corresponding 95% CI was very close to 100%, indicating no difference with regard to ANC after administration of the Pelmeg and Neulasta. Intrasubject CV was low, with 7.49%.

**Table 3:** Statistical analysis of primary PD parameter AUEC_0‐last_ of ANC (model‐based PD set, N = 82)

Pelmeg/Neulasta		
Ratio (%)	95% CI	Intra‐subject CV (%)^a^
101.59	99.58; 103.63	7.49

Abbreviations: ANC = absolute neutrophil count, AUEC_0‐last_ = area under the effect time curve from time zero to last available concentration, CI = confidence interval, CV = coefficient of variation, N = number of subjects, PD = pharmacodynamic.

a Intraindividual CV (%) estimated from the residual mean squares.

Of note, when using tighter acceptance limits (90.00%‐111.00%), comparability between test and reference with regard to PD was shown as well. This underlines the high degree of similarity between Pelmeg and Neulasta with regard to PD.

Overall, the primary PD endpoint of this study was met and PD comparability between test and reference was shown.

#### 3.2.1 Descriptive analysis of secondary PD endpoints

A descriptive summary of the PD parameters for ANC, based on the PD set, is shown in Table 4.

**Table 4:** Summary of PD parameters of ANC (PD Set, N = 93)

Parameter	Neulasta		Pelmeg	
	N = 93		N = 93	
AUEC_0‐last_ [h*G/L]	6173.3/22.1	n = 131	6207.9/24.1	n = 129
*E*_max_ [G/L]	28.4/25.4	n = 131	28.4/23.9	n = 129
*t*_max, E_ [h]	36.0 (24.0‐84.0)	n = 131	36.0 (12.0‐84.0)	n = 129

Note

Geometric mean/CV(%) are presented for AUEC_0‐last_ and *E*_max_, median and range for *t*_max, E_. Please note that due to the partial replicate design of the study, each subject is contributing data from two periods for one treatment and data from one period for the other treatment (leading to numbers for “n” that are larger than the number of subjects “N”).

Abbreviations: ANC, absolute neutrophil count; AUEC_0‐last_, area under the effect time curve from time zero to last available concentration; CV, coefficient of variation; *E*_max_, maximum effect; n, number of subjects’ periods contributing to the calculation of the descriptive statistics; N, number of subjects in group; PD, pharmacodynamic; *t*_max,E_, time of maximum effect.

Geometric mean AUEC_0‐last_ and *E*_max_ were similar after administration of Neulasta and Pelmeg. Median *t*_max_, *E* was 36 hours after both treatments.

Similar increases in CD34^+^ cells were seen after administration of Neulasta and Pelmeg. Values peaked at around 5 days postdose, and nearly reached predose levels at the last sampling point on Day 10 postdose.

### 3.3 Immunogenicity

Immunogenicity was assessed as primary endpoint. For the analysis of ADAs, the subject who received an incorrect treatment sequence (TRR) was excluded, as this sequence was incompatible with the intended study design, that is, repeated administrations of the same treatment in Period 1 and 2. The respective subject with the incorrect treatment sequence was ADA negative. Thus, the analysis was based on 95 subjects.

No filgrastim reactivity and no neutralizing antibodies were detected. Overall, two confirmed ADA positive samples were detected, both occurring at Day 15 of Period 1. One subject dosed with Neulasta was positive for Pelmeg, Neulasta, and PEG, and one subject dosed with Pelmeg was positive for Neulasta. These samples had a low ADA titer, no filgrastim reactivity, and no neutralizing capacity. Also, no impact on PK or PD was detected in subjects with ADA positive samples.

The primary immunogenicity endpoint was proportion of ADA‐positive subjects at the end of Period 2, as detected by a confirmatory assay. For the primary analysis, proportions of ADA‐positive subjects at the end of Period 2 were calculated and presented with corresponding 95% CIs per treatment. Furthermore, the difference of proportions of ADA‐positive subjects between treatments was calculated and presented with corresponding 95% CIs. Results are shown in Table 5.

**Table 5:** Frequency distribution of ADA‐positive subjects ‐ ADA‐positive cumulative (safety set, N = 95^a^)

Treatment sequence						Treatment comparison	
RRT (N = 47)			TTR (N = 48)			Difference T‐R (%)	95% CI for the difference
n (%)	n#	95% CI	n (%)	n#	95% CI		
1 (2.1)	44	0.06%; 12.02%	1 (2.1)	46	0.06%; 11.53%	0	‐9.96%; 9.40%

Note

For 95% CI only number of subjects with an available result per scheduled study time were used.

Abbreviations: R, reference treatment (Neulasta); T, test treatment (Pelmeg); CI, confidence interval, n#, number of available results.

a The subject treated with an incorrect sequence was excluded.

No difference in immunogenicity between Pelmeg and Neulasta was observed.

### 3.4 Pharmacokinetics

The PK parameters AUC_0‐120 h_, *C*_max_, and *t*_max_ were analyzed purely descriptively. Geometric mean AUC_0‐120 h_ and geometric mean *C*_max_ were similar for Neulasta and Pelmeg (AUC_0‐120 h_: 821.8 and 847.2 h*ng/mL, *C*_max_: 29.5 and 29.9 ng/mL). Variability was high with geometric CVs of 116%‐124% for AUC_0‐120 h_ and 133%‐143% for *C*_max_. Median *t*_max_ occurred at 12 hours after both treatments.

Overall, the results were found to be very similar for Pelmeg and Neulasta.

### 3.5 Safety

All 96 subjects dosed were included in the safety analysis. The percentage of subjects with any AE was comparable for Pelmeg and Neulasta (79.2% vs 83.3%, Table 5). In both groups, most AEs were deemed drug related by the investigator. In most subjects, AEs were of mild or moderate severity. There were no deaths. Three subjects experienced serious adverse events (SAEs) during the ambulatory phase of the study; one subject with local swelling after a cosmetic intervention, which was not permitted per protocol (following treatment with Neulasta), two subjects with influenza (one each following treatment with Neulasta and Pelmeg). None of the SAEs was assessed as related to study drug. Four subjects discontinued due to AEs; two of these after Pelmeg (alanine aminotransferase [ALT] increased, lower back pain), and two of these after Neulasta (ALT increased, blood pressure increased).

The pattern of AEs was similar for Pelmeg and Neulasta, with most patients experiencing AEs in the System Organ Class (SOC) of musculoskeletal and connective tissue disorders. Most commonly reported preferred terms (PTs) after both treatments were back pain, headache, nasopharyngitis, hypoglycemia, and pain in extremity. Safety results are summarized in Table 6.

**Table 6:** Summary of safety results (safety set, N = 96)

Subjects with AE, n (%)	Neulasta	Pelmeg	Total
Any AE	80 (83.3)	76 (79.2)	92 (95.8)
Drug‐related AE	73 (76.0)	71 (74.0)	89 (92.7)
Serious AE	2 (2.1)	1 (1.0)	3 (3.1)
AE leading to discontinuation	2 (2.1)	2 (2.1)	4 (4.2)
Deaths	0 (0)	0 (0)	0 (0)
AEs by severity			
Mild	66 (68.8)	67 (69.8)	86 (89.6)
Moderate	52 (54.2)	60 (62.5)	76 (79.2)
Severe	2 (2.1)	5 (5.2)	7 (7.3)
Most common AEs by Preferred Term (≥2% of subjects in any group)			
Back pain	57 (59.4)	50 (52.1)	75 (78.1)
Headache	22 (22.9)	29 (30.2)	40 (41.7)
Nasopharyngitis	19 (19.8)	23 (24.0)	36 (37.5)
Hypoglycemia	20 (20.8)	21 (21.9)	29 (30.2)
Pain in extremity	10 (10.4)	9 (9.4)	16 (16.7)
Blood pressure systolic increased	9 (9.4)	9 (9.4)	14 (14.6)
Arthralgia	4 (4.2)	7 (7.3)	11 (11.5)
Nausea	1 (1.0)	8 (8.3)	9 (9.4)
Cough	1 (1.0)	6 (6.3)	7 (7.3)
Blood creatine phosphokinase increased	1 (1.0)	5 (5.2)	6 (6.3)
Oropharyngeal pain	1 (1.0)	5 (5.2)	6 (6.3)
Musculoskeletal pain	2 (2.1)	3 (3.1)	5 (5.2)
C‐reactive protein increased	4 (4.2)	2 (2.1)	5 (5.2)
Blood pressure increased	4 (4.2)	4 (4.2)	5 (5.2)
Chest pain	2 (2.1)	3 (3.1)	5 (5.2)
Myalgia	3 (3.1)	3 (3.1)	4 (4.2)
Alanine aminotransferase increased	3 (3.1)	1 (1.0)	4 (4.2)
Toothache	1 (1.0)	4 (4.2)	4 (4.2)
Vomiting	0 (0.0)	4 (4.2)	4 (4.2)
Gastroenteritis	2 (2.1)	1 (1.0)	3 (3.1)
Gamma glutamyltransferase increased	1 (1.0)	3 (3.1)	3 (3.1)
Aspartate aminotransferase increased	1 (1.0)	2 (2.1)	3 (3.1)
Diarrhea	1 (1.0)	2 (2.1)	3 (3.1)
Abdominal pain	0 (0.0)	3 (3.1)	3 (3.1)
Hematuria	1 (1.0)	2 (2.1)	3 (3.1)
Neck pain	1 (1.0)	1 (1.0)	2 (2.1)
Dizziness	1 (1.0)	1 (1.0)	2 (2.1)
Paresthesia	2 (2.1)	0 (0.0)	2 (2.1)
Influenza	1 (1.0)	1 (1.0)	2 (2.1)
Hyperkalemia	1 (1.0)	1 (1.0)	2 (2.1)
Blood bilirubin increased	2 (2.1)	0 (0.0)	2 (2.1)
Chest discomfort	1 (1.0)	1 (1.0)	2 (2.1)
Pyrexia	0 (0.0)	2 (2.1)	2 (2.1)
Discomfort	1 (1.0)	1 (1.0)	2 (2.1)
Feeling cold	0 (0.0)	2 (2.1)	2 (2.1)
Puncture site pain	2 (2.1)	0 (0.0)	2 (2.1)
Arthropod sting	1 (1.0)	1 (1.0)	2 (2.1)
Ocular hyperemia	1 (1.0)	1 (1.0)	2 (2.1)
Hematoma	1 (1.0)	1 (1.0)	2 (2.1)

Note

Percentages are based on N. AEs were coded using the Medical Dictionary for Regulatory Activities (MedDRA) version 19.1.

Abbreviations: AE, adverse event; N, number of subjects.

Injection site reactions were reported for one subject after administration of Pelmeg (injection site hematoma), and for three subjects after administration of Neulasta (one event of hematoma and two events of puncture site pain). Injection site reactions were assessed as mild in all subjects.

No clinically meaningful differences between treatments were observed for any safety assessments, including laboratory, ECG, or vital signs.

## 4 DISCUSSION

The focus of this clinical study was to confirm the findings of similar immunogenicity between Pelmeg and Neulasta seen in the pivotal PK/PD study B12019‐101, after multiple dosing with study drug, and to further confirm the biosimilarity for the PD endpoint, at a dose that is the ascending part of the dose‐response curve.^8^, ^9^

For the primary PD endpoint comparability of Pelmeg and Neulasta was shown. Of note, PD comparability was also demonstrated when applying a tighter acceptance interval of 90.00%‐111.00% (as suggested by the Draft EMA Guideline on similar biological medicinal products containing recombinant granulocyte‐colony stimulating factor, EMEA/CHMP/BMWP/31329/2005 Rev 1, July 2018).

No filgrastim reactivity and no neutralizing antibodies were detected. Two confirmed ADA positive samples were detected, one each after treatment with Pelmeg and Neulasta, respectively. These had a low ADA titer, no filgrastim reactivity, and no neutralizing capacity. Also, no impact on PK or PD was detected in subjects with ADA positive samples. This is in line with postmarketing experience for both pegfilgrastim and filgrastim, which has demonstrated an absence of clinically impactful immunogenicity associated with the use of either product, even in fully immune competent populations. The literature reports results from a prospective 5‐year study of 6768 peripheral blood stem cell donors who were treated with G‐CSF and 2726 bone marrow donors who were not treated with G‐CSF.^13^ The results of that study showed that peripheral blood stem cell donors were not at increased risk for developing an autoimmune disease when compared to bone marrow donors. In addition, the US FDA has stated that they are unaware of reports of neutralizing antibodies to G‐CSF products, concluding that the literature indicates that G‐CSF products are low risk for causing ADA‐related severe adverse effects (FDA, Transcript of FDA Adcom for Zarxio^14^). The safety data set for Pelmeg was reviewed in detail for AEs that could potentially be immune‐mediated, with an emphasis on hypersensitivity reactions. There were no AEs classified as hypersensitivity or drug hypersensitivity in subjects treated with either Pelmeg or Neulasta, and local tolerability was good.

In this study, subjects were administered three doses of study drug T‐T‐R or R‐R‐T), resulting in an overall exposure time of approximately 18 weeks. In comparison, in pivotal studies leading to the regulatory approval of Neulasta, cancer patients were exposed to a mean of 3.8 injections of Neulasta at doses of 30, 60, or 100 μg/kg, or a fixed dose of 6 mg,^15^ In these pivotal studies, chemotherapy was repeated every 3 weeks for up to four cycles, resulting in a total exposure time of approximately 12 weeks. This illustrates the similarity in exposure time between our study and exposure time typically seen in the target population of cancer patients. In clinical practice, it seems common to administer G‐CSFs for relatively short courses and not from the first cycle of chemotherapy on.^16^

The safety profile of Pelmeg was characterized by AEs that are known adverse drug reactions of Neulasta, mainly musculoskeletal disorders and headache. Thereby, the frequencies and pattern of AEs were similar between Pelmeg and Neulasta, and in line with the product information for Neulasta. Drug‐related hypoglycemia was reported in around 20% of subjects after administration of Pelmeg and Neulasta. Of note, all events of hypoglycemia were transient, asymptomatic and did not require medical intervention. The high frequency of hypoglycemia is attributed to the conservative reporting approach for laboratory AEs in this study, and not considered of clinical relevance. No clinically remarkable differences between Pelmeg and Neulasta have been reported with respect to clinical laboratory parameters, vital signs and cardiovascular safety.

## 5 CONCLUSION

Results from this study have confirmed PD comparability of Pelmeg and Neulasta at a dose of 3 mg. No meaningful differences in immunogenicity or safety were observed. The results from this supportive study, in combination with the results from the pivotal PK/PD study (B12019‐101) confirmed the overall biosimilarity of Pelmeg and Neulasta, and have led to the regulatory approval of Pelmeg in the EU in 2018.

## DISCLOSURE

This study was funded by Cinfa Biotech, now part of the Mundipharma network of independent associated companies. KR, HW, and RJ are employees of Cinfa Biotech, now part of Mundipharma. JH is an employee of Staburo GmbH. DL is an employee of the University of Lucerne.

## AUTHOR CONTRIBUTIONS

KR, HW, DL, JH, and RJ contributed to the conception and design of the study. DL and JH contributed to the statistical analysis of the data. All authors contributed to the interpretation of the data. All authors contributed to drafting of the manuscript, revised the manuscript critically for intellectual content, and approved the final submitted version.

Pelmeg^®^ is a registered trademark of Cinfa Biotech, SL (a member of the Mundipharma network of independent associated companies). Neulasta^®^ is a registered trademark of Amgen.
